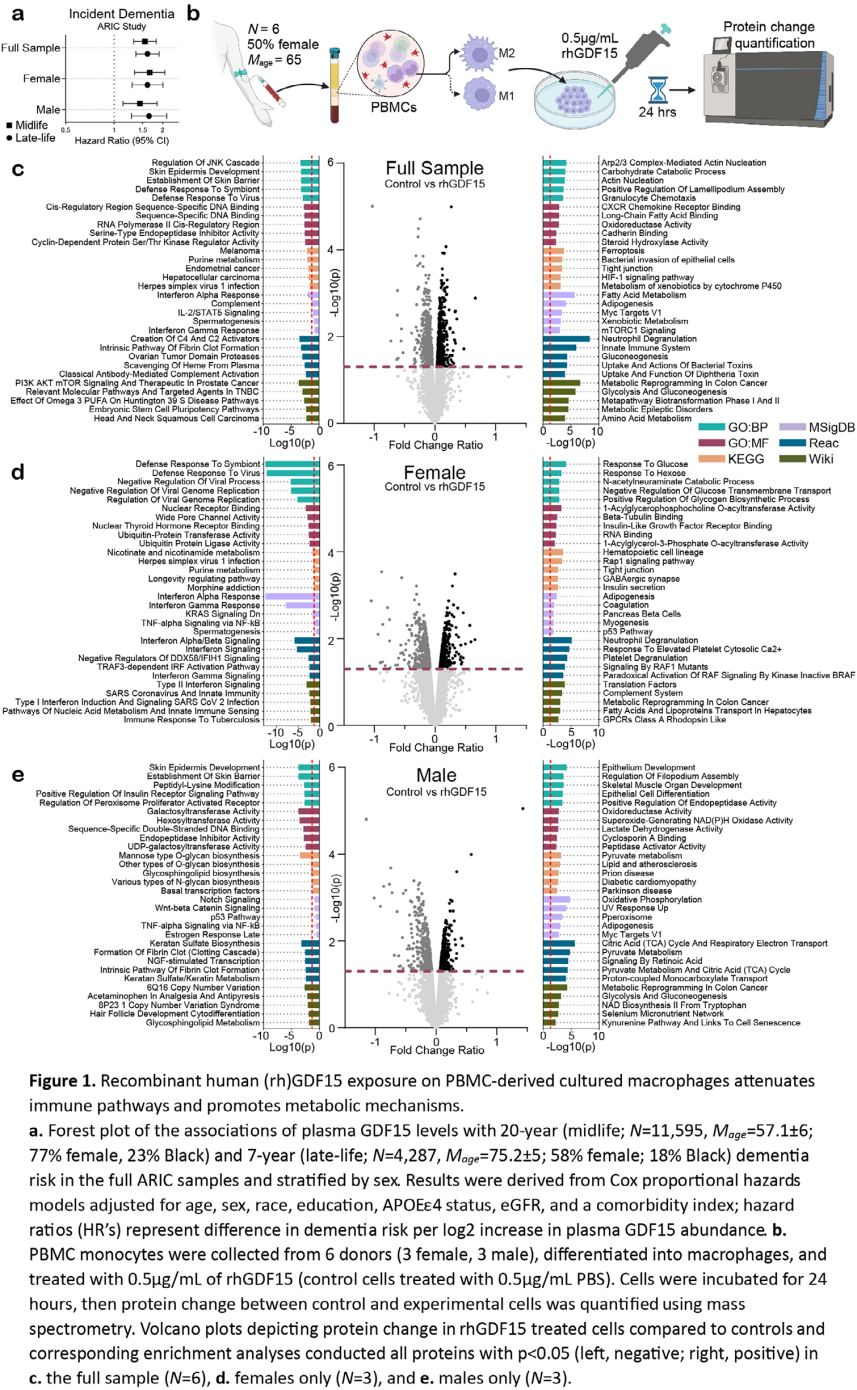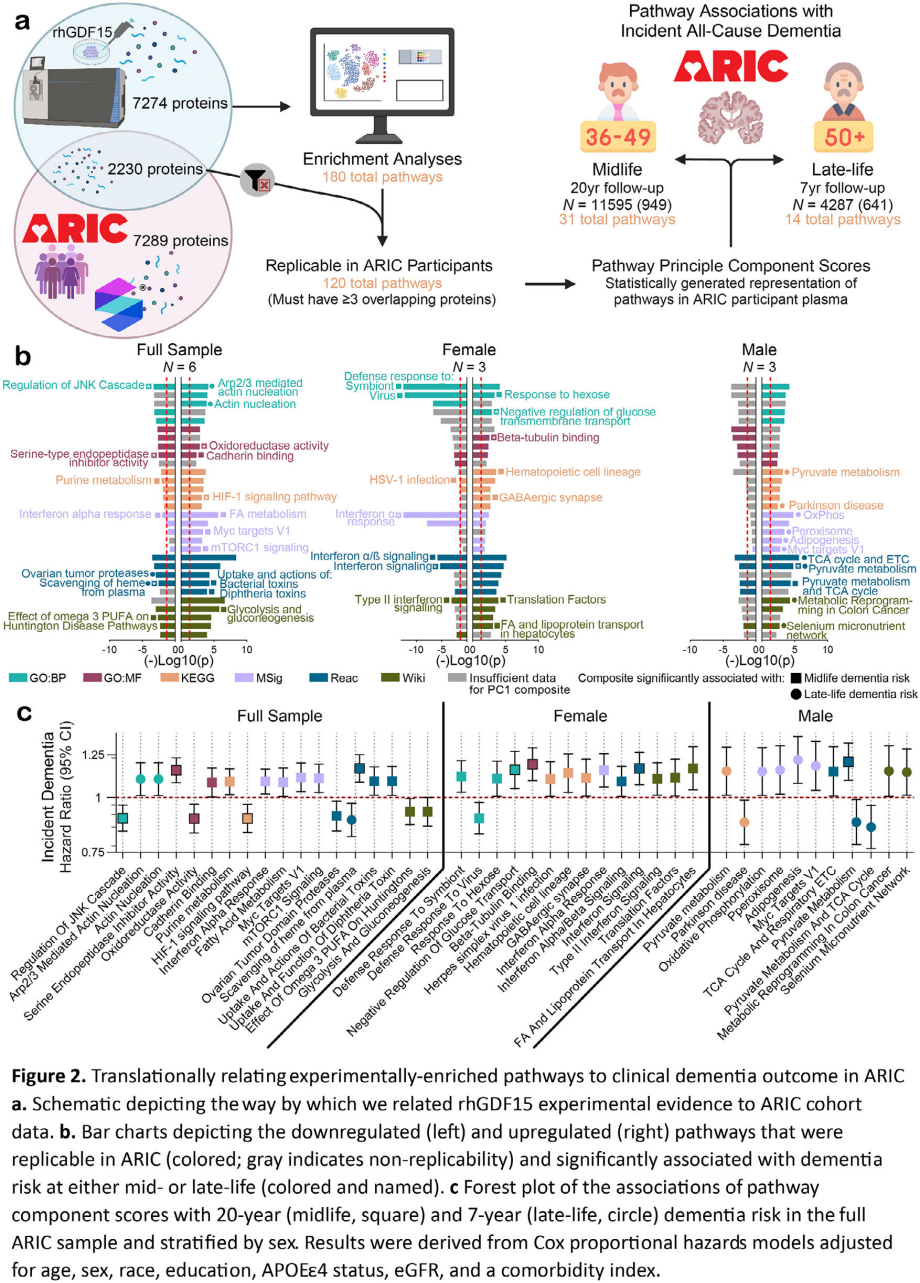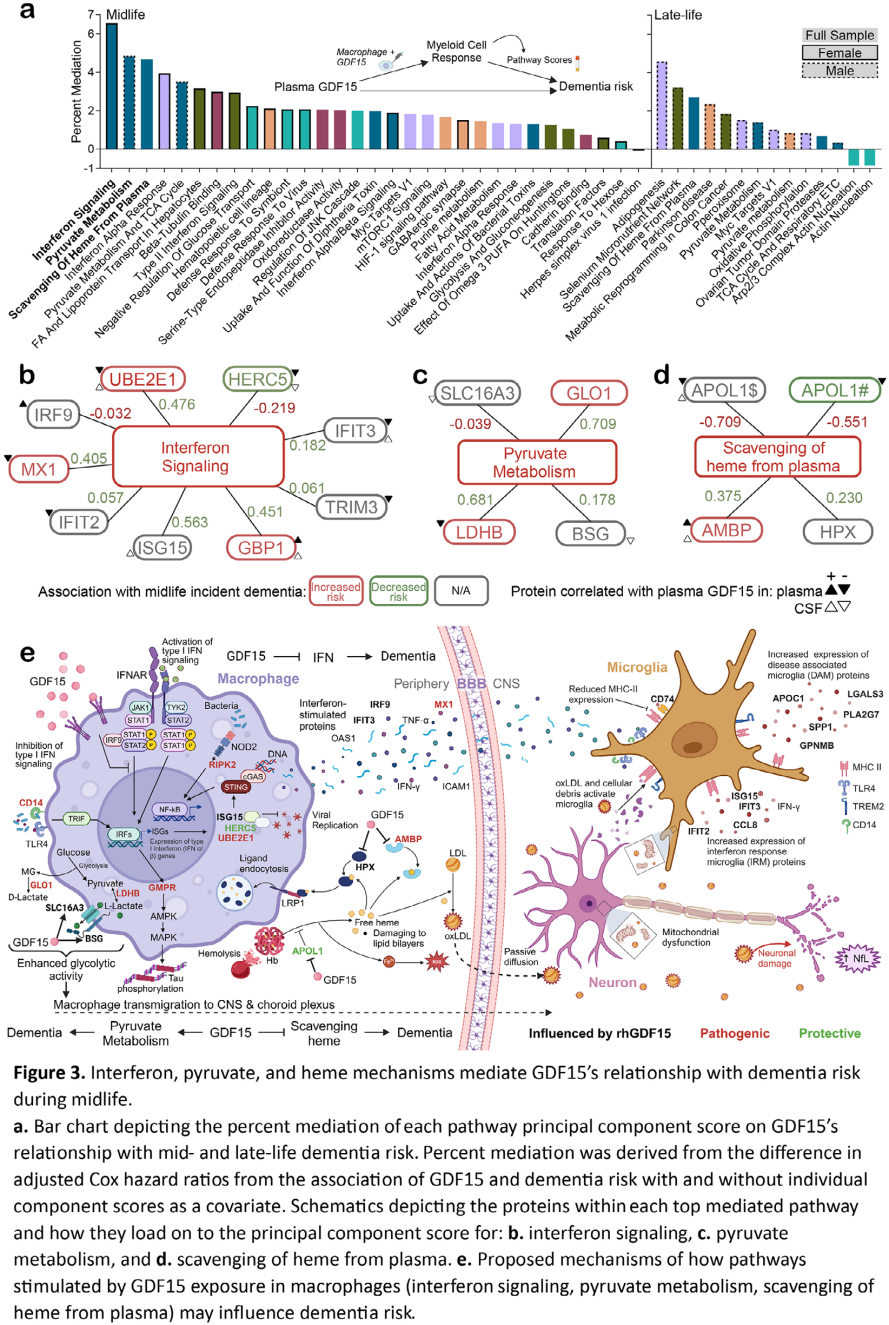# Understanding the mechanisms linking GDF15 to dementia risk: A translational approach

**DOI:** 10.1002/alz70855_106820

**Published:** 2025-12-24

**Authors:** Cassandra O Blew, Michael R. Duggan, Dimitrios Tsitsipatis, Alison B Herman, Cassandra M Joynes, Jingsha Chen, Mary Kaileh, Ranjan Sen, Aditi Gupta, Krystyna Mazan‐Mamczarz, Myriam Gorospe, Alexey Lyashkov, Yevgeniya Lukyanenko, Rebecca F. Gottesman, Christie M Ballantyne, Michael E. Griswold, Keenan A. Walker

**Affiliations:** ^1^ Laboratory of Behavioral Neuroscience, National Institute on Aging, Intramural Research Program, Baltimore, MD, USA; ^2^ Vascular Aging Biology Unit, National Institute on Aging, Intramural Research Program, Baltimore, MD, USA; ^3^ Translational Senescence Unit, National Institute on Aging, Intramural Research Program, Baltimore, MD, USA; ^4^ Department of Epidemiology, Johns Hopkins University Bloomberg School of Public Health, Baltimore, MD, USA; ^5^ Laboratory of Molecular Biology and Immunology, National Institute on Aging, National Institutes of Health, Baltimore, MD, USA; ^6^ Laboratory of Genetics and Genomics, National Institute on Aging (NIA) Intramural Research Program (IRP), National Institutes of Health (NIH), Baltimore, MD, USA; ^7^ Translational Gerontology Branch, National Institute on Aging, National Institutes of Health, Baltimore, MD, USA; ^8^ Laboratory of Cardiovascular Science, National Institute of Aging, National Institutes of Health, Baltimore, MD, USA; ^9^ National Institute of Neurological Disorders and Stroke, Intermural Research Program, National Institutes of Health, Bethesda, MD, USA; ^10^ Baylor College of Medicine, Houston, TX, USA; ^11^ University of Mississippi Medical Center, The MIND Center, Jackson, MS, USA

## Abstract

**Background:**

Our group previously identified higher growth/differentiation factor‐15 (GDF15) abundance in plasma among individuals who developed dementia (Figure 1a), which was accompanied by altered expression of immune proteins in plasma and cerebrospinal fluid. However, whether GDF15 is a valid dementia risk factor, and the biologic mechanisms by which GDF15 may influence dementia incidence, remain unknown.

**Methods:**

Using macrophages differentiated from PBMC‐isolated monocytes from six donors (50% female, *M_age_* = 65), we examined the proteomic changes (via mass spectrometry) of cells exposed to 0.5µg/mL recombinant human GDF15 (rhGDF15) compared to controls (Figure 1b). We performed enrichment analyses of the rhGDF15‐macrogphage proteome to identify the top up‐ and down‐regulated biological pathways. Using pathway annotations and overlapping proteins measured in the Atherosclerosis Risk in Communities (ARIC) study (SomaScan assay v4.0), we re‐created these pathways in *N* = 11,595 middle‐aged (*M_age_* = 57±6) and *N* = 4,287 older adults (*M_age_* = 75±5) (Figure 2a). Scores derived from the first principal component for each experimentally‐enriched pathway were associated with 20‐year (for midlife) and 7‐year (for late‐life) dementia risk using Cox regression. Analyses were conducted in the full sample and sex‐stratified subsets.

**Results:**

Enrichment of macrophage proteins altered by rhGDF15 exposure revealed an upregulation of metabolic pathways and a downregulation of inflammatory responses in both the full sample and sex‐stratified analyses (Figure 1c‐e). Of the 180 rhGDF15‐enriched pathways identified by mass spectrometry, 120 were re‐created using SomaScan proteomic data in ARIC. We found 31 and 14 pathway composite scores were associated with midlife and late‐life dementia risk, respectively (Figure 2a‐c). Secondary analyses revealed that during midlife, *Interferon signaling, Pyruvate metabolism*, and *Scavenging of heme from plasma* were the strongest biological mediators, accounting for 6.56%, 4.86%, and 4.67% of GDF15's relationship with incident dementia, respectively (Figure 3a). By analyzing the proteins within these three pathways (Figure 3b‐d), we propose mechanisms by which GDF15's proteo‐biological signature may contribute to the development of dementia (Figure 3e).

**Conclusions:**

GDF15's relationship with dementia risk may be explained by its effects on macrophages and other peripheral myeloid cells. By attenuating interferon signaling and heme scavenging and increasing pyruvate metabolism, plasma GDF15 may increase dementia risk.